# Clinical Application Progress of Artificial Intelligence in Pancreatic Cancer: From Diagnosis to Immunotherapy

**DOI:** 10.32604/or.2026.078793

**Published:** 2026-06-16

**Authors:** Zehao Wei, Xuejian Liu, Zheng Zhang, Yimin Ma, Min Xu

**Affiliations:** 1 Department of Gastroenterology, Affiliated Hospital of Jiangsu University, Jiangsu University, Zhenjiang, China; 2 Department of Radiology, Xinjiang 474 Hospital, Urumqi, China

**Keywords:** Pancreatic cancer, artificial intelligence (AI), immunotherapy, tumor microenvironment (TME)

## Abstract

Pancreatic cancer is one of the most lethal malignancies, characterized by difficulties in early diagnosis, limited therapeutic options, and generally poor patient prognosis. In recent years, immunotherapy has provided new opportunities for the treatment of pancreatic cancer; however, its clinical efficacy has been substantially constrained by the complex tumor microenvironment (TME) and immune evasion mechanisms. With the rapid advancement of artificial intelligence (AI) technologies, AI has demonstrated great potential in the early detection of pancreatic cancer, prediction of immunotherapeutic responses, and design of personalized treatment strategies. This review systematically summarizes the latest advances in the application of artificial intelligence in pancreatic cancer immunotherapy, with a particular focus on key AI assisted technologies, including tumor immune microenvironment characterization, prediction of genetic mutation profiles, nanomedicine design, and dynamic monitoring of therapeutic responses. By integrating single cell sequencing and multi-omics data analyses, we discuss how AI can effectively address critical bottlenecks in immunotherapy. In addition, this article analyzes current technical challenges and future development trends, aiming to provide a theoretical foundation and practical guidance for achieving precision immunotherapy in pancreatic cancer and to promote clinical translation and application in this field.

## 1 Introduction

Pancreatic cancer is a highly malignant disease with extremely poor outcomes. It is associated with a very high death rate. In addition, many patients are diagnosed at an advanced stage, which greatly limits treatment options. As a result, this disease remains a serious challenge for clinical practice and public health worldwide [1,2,3]. Pancreatic cancer has several unfavorable clinical features. Its early symptoms are usually mild and difficult to recognize. The deep anatomical location further delays detection. In addition, the disease is highly aggressive and shows limited sensitivity to standard therapies, including operative and drug based approaches. Many patients are therefore diagnosed at an advanced stage or already have distant spread, which limits effective clinical intervention [4]. Together, these issues lead to a consistently poor long term prognosis in patients with this disease. The overall survival at five years remains very low, with reported values of about 7%–12% [2,5,6]. At present, pancreatic cancer is mainly identified using medical imaging techniques. Hybrid PET/MRI systems integrate structural detail from MRI and functional information from PET. This combination improves performance in early screening, disease assessment, and treatment evaluation. It also offers basic guidance for clinical decision making [7]. Liquid biopsy based biomarker research has gained increasing attention. Blood derived indicators, such as ctDNA, miRNAs, and extracellular vesicles, show strong value for early testing without invasive procedures and for outcome assessment. Under the complex conditions of the tumor microenvironment (TME), these methods offer new advantages compared with conventional markers. They may support earlier identification of disease and improve patient management in pancreatic cancer [8,9,10,11].

In recent years, immune based therapies have become an important advance in oncology. These approaches act by stimulating the body’s defense system to target cancer cells. Clear benefits have been reported in several solid cancers, for example melanoma and lung malignancies. In contrast, pancreatic cancer responds poorly to this type of treatment. This low response is largely linked to a strongly suppressive local tumor environment. The pancreatic TME contains extensive fibrous tissue and a large number of activated fibroblasts. It also includes many suppressive immune cell types, such as T regulatory cells, macrophages with tumor supporting functions, and myeloid suppressor cells. Together, these elements limit the entry of effective immune cells and support tumor escape through multiple signaling routes [12,13,14]. Certain patients with pancreatic cancer benefit from therapies that block immune inhibitory pathways, including programmed cell death protein 1 (PD-1) or programmed cell death-Ligand 1 (PD-L1) targeting agents. This benefit is mainly observed in cases with microsatellite instability or defects in mismatch repair. However, this subgroup accounts for only a small proportion of all patients. Therefore, applying immunotherapy broadly in pancreatic cancer remains difficult [15]. Current studies mainly explore combined immunotherapy, treatments targeting multiple pathways, and strategies that modify the TME. These approaches aim to make immunotherapy more effective and help patients live longer [16,17,18]. In addition, nanotechnology and exosome based systems have emerged as novel drug delivery platforms and immunomodulatory tools, offering new opportunities for pancreatic cancer immunotherapy by improving tumor targeting and immune activation, thereby further enhancing therapeutic outcomes [19,20].

Over the past few years, progress in artificial intelligence (AI) based methods has strongly influenced cancer care. These tools support earlier disease identification, outcome prediction, and individualized treatment planning. They also open new directions for managing pancreatic cancer [21]. AI shows strong performance when handling diverse omics information. This includes imaging features, tissue based data, and genetic profiles. Such approaches are effective for large scale data analysis and pattern discovery. They help clinicians classify disease more precisely and estimate patient outcomes more reliably [22,23,24]. In pancreatic cancer research, AI tools support rapid early screening by examining imaging results and liquid biopsy information. They also combine immune related and molecular signals to estimate how patients may respond to immunotherapy, including immune checkpoint inhibitor (ICI) based approaches. This supports the design of more individualized therapeutic plans [21,25,26]. AI based omics analysis tools support the planning and assessment of combined treatment approaches. Learning based methods also help analyze complex biological interactions within tumors. These techniques provide useful insights into immune escape processes and local tumor environments in this disease [27].

In conclusion, immunotherapy shows meaningful potential in this disease, but its clinical benefit is still restricted by a complex local tumor environment and immune escape pathways. This review provides an overview of recent progress where AI methods meet immunotherapy research. It also discusses key strengths and current limitations. In addition, future directions are outlined with a focus on cross field collaboration and new treatment development. Together, these points aim to offer conceptual and technical support for continued work in this fast growing area.

## 2 Immune Microenvironment of Pancreatic Cancer

The TME of pancreatic cancer is highly complex and strongly immunosuppressive, representing a key factor driving malignant progression and therapeutic resistance, including immune tolerance and immune evasion. This microenvironment forms both physical and immunological barriers that impede effective immune cell infiltration and antitumor immune responses.

### 2.1 Imbalanced Immune Cell Populations

Cells with inhibitory immune functions are key components of the local tumor environment in this disease. One major group is regulatory T lymphocytes, which weaken antitumor immunity through the release of inhibitory signaling molecules, including IL-10 and TGF-β. They also reduce the activity of effector T cells through direct contact. Another important population is myeloid suppressor cells. These cells limit T-cell responses by generating oxidative and nitrogen related metabolites, which further strengthen immune suppression within the tumor site [28]. Cancer associated fibroblasts (CAFs) represent a dominant stromal cell type in this disease. They release many signaling factors that attract macrophages with suppressive features and regulatory T cells. These fibroblasts also generate large amounts of extracellular matrix (ECM) components, including different collagens and hyaluronic acid. This activity leads to a compact matrix structure and increases both physical and immune barriers within the local tumor environment [29,30,31].

### 2.2 Complex Physical Barriers

Structural barriers in this disease are largely shaped by disordered blood vessels and excessive matrix deposition. Tumor tissues receive limited blood supply, which leads to long lasting oxygen deficiency. Low oxygen conditions activate hypoxia related signaling pathways. These signals increase resistance to treatment and drive changes in cell metabolism, stem like traits, invasive behavior, and abnormal vessel formation [1,6]. The extracellular matrix raises pressure within the tissue. It also restricts the entry of both immune effector cells and therapeutic compounds. Furthermore, dense stromal components, such as collagen fibers and fibronectin, form mechanical barriers that hinder immune cell penetration into the tumor core [32,33]. Collectively, these physical factors contribute to poor immune cell infiltration in pancreatic cancer, resulting in an immunologically “cold” tumor.

### 2.3 Multiple Immune Evasion Mechanisms

At the molecular scale, escape from immune control in this disease is linked to genetic alterations and disrupted signaling within tumor cells. Mutations in Kirsten rat sarcoma viral oncogene (KRAS) occur frequently and alter cellular metabolic activity. They also reshape local immune conditions around the tumor. These changes weaken antigen display and increase the expression of inhibitory surface molecules, including PD-L1, which supports immune resistance [34]. High levels of MYC have been found to control several immune suppressing processes. One effect is the increase in polyamine metabolism, which strongly influences the behavior of macrophages near the tumor and promotes the growth of suppressive myeloid cells. These changes further reduce T-cell activation [35]. In addition, an imbalance in the ratio of CD8-positive effector T cells (Teffs) to FOXP3-positive regulatory T cells (Tregs) suppresses the immune status of pancreatic ductal adenocarcinoma (PDAC) [36]. Pancreatic cancer usually has a low level of tumor mutations. This leads to a smaller number of tumor specific antigens and weakens the ability of the immune system to recognize the tumor [37]. Tumor cells can alter immune related gene activity through epigenetic mechanisms. For example, WDR5 dependent H3K4me3 modifications influence how antigens are presented and regulate pathways that suppress immune responses [30]. These molecular mechanisms synergistically establish a highly immunosuppressive TME in pancreatic cancer, which profoundly impacts the efficacy of immunotherapy.

**Figure 1:**
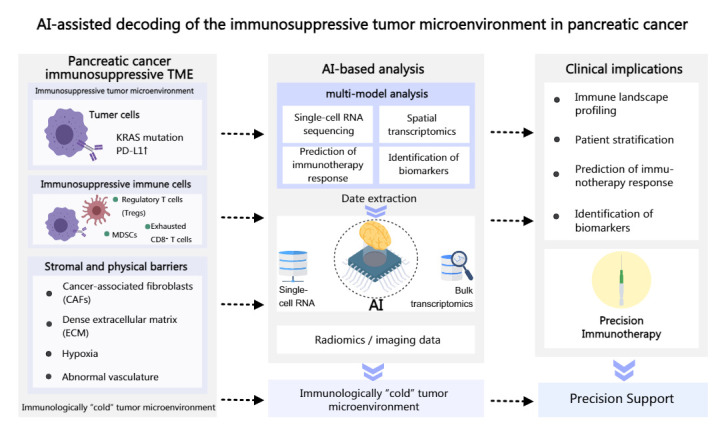
**Artificial intelligence (AI) assisted decoding of the tumor microenvironment in pancreatic cancer.** Pancreatic cancer is characterized by a highly immunosuppressive tumor microenvironment (TME) composed of Kirsten rat sarcoma viral oncogene (KRAS) mutant tumor cells, regulatory T cells, myeloid derived suppressor cells, exhausted CD8^+^ T cells, and dense stromal components formed by cancer associated fibroblasts and extracellular matrix deposition. Hypoxia and abnormal vasculature further limit immune cell infiltration. AI enables integrative analysis of multi-modal data to characterize TME heterogeneity and spatial organization, supporting immune profiling, patient stratification, and prediction of immunotherapy response in pancreatic cancer (The figures were created using MedPeer).

Overall, immune suppression in pancreatic tumors involves multiple layers. This includes the activity of inhibitory immune cells, the development of physical barriers such as abnormal blood vessels and a matrix rich in hyaluronic acid, and mutations that allow tumor cells to evade immune detection. AI enables integrative analysis of multi-modal data, patient classification, and the estimation of individual immunotherapy responses for this disease (Fig. 1).

## 3 Applications of AI Assisted Multi-Omics in Early Detection and Therapeutic Prediction of Pancreatic Cancer

### 3.1 Applications of AI in Imaging Based Diagnosis

The insidious progression of pancreatic cancer and the lack of obvious morphological abnormalities in its early stages make early detection and diagnosis challenging. Traditional imaging methods, such as contrast enhanced CT, are still widely used for diagnosis. However, their accuracy depends on the skill of the radiologist and can miss small or subtle abnormalities. Recently, AI approaches, especially deep learning and radiomics, have improved imaging based detection of this disease. By automatically extracting structural features and subtle differences that are difficult to detect with the naked eye from large scale imaging datasets, AI can identify early signals of pancreatic ductal adenocarcinoma (PDA) even before clinical manifestations, thereby improving early detection rates. AI based methods for segmenting the pancreas improve consistency between analyses. They also help detect imaging features that indicate early tumor development [38].

Deep learning algorithms applied to high dimensional imaging data, particularly convolutional neural networks (CNNs) and transformer models, can automatically extract complex morphological and metabolic features of the pancreas at both local and whole organ levels, enabling highly sensitive detection of early or occult lesions. Current AI models are capable of identifying subtle histological changes in different pancreatic subregions, distinguishing healthy tissue from precancerous lesions, and effectively predicting the risk of pancreatic cancer, this performance is validated through a retrospective study involving 108 contrast enhanced abdominal CT scans (from 72 subjects split into internal and external datasets) and external testing on 28 independent scans (14 healthy controls, 14 pre-diagnostic cases), achieving an accuracy of approximately 89.3%, sensitivity of 86%, and specificity of 93% [39]. Combining different imaging methods, including CT, MRI, and endoscopic ultrasound, with AI analysis improves diagnostic accuracy. This approach also supports earlier detection and may allow real time use of imaging markers for screening and predicting patient outcomes [40,41]. In addition, AI has shown excellent performance in automated pancreatic image segmentation, lesion localization, and classification tasks, significantly improving the precision of quantitative imaging analysis and its clinical utility [42,43].

Endoscopic ultrasound (EUS) is an important method for identifying pancreatic tumors. Its diagnostic reliability, however, varies with the operator’s level of expertise. The introduction of AI helps reduce this dependence and supports earlier disease identification. A meta-analysis of 7 studies involving 1110 participants (634 pancreatic cancer cases and 476 non-cancer cases) and multiple independent validations report that deep learning models show strong diagnostic ability, with Area Under Curve (AUC) values reaching 0.95 (95% CI, 0.93–0.97) [44,45]. Sensitivity and specificity as high as 93% and 90% have also been observed, which are clearly higher than those achieved by non-expert clinicians. These performance metrics are supported by both internal cross validation and external validation using independent datasets (e.g., 1000 EUS images in external testing) [44,45]. AI assisted systems not only enhance the objectivity and reliability of diagnoses but also improve physicians’ diagnostic skills, reducing rates of misdiagnosis and missed diagnoses. Moreover, AI models have shown strong performance in differentiating pancreatic cancer from imaging mimicking conditions such as chronic pancreatitis and autoimmune pancreatitis, providing valuable clinical decision support. In a prospective non-randomized controlled trial, 308 patients with suspected pancreatic lesions or at high risk for pancreatic cancer were enrolled to evaluate the solid lesion detection and segmentation performance of the AI-enhanced endoscopic ultrasound (AI-EUS) system, PANCRAIEUS. Using conventional EUS performed by experts as the gold standard, AI-EUS achieved a solid lesion detection rate of 97.1%, which was not significantly different from the 100% detection rate of conventional EUS (*p* = 0.25), indicating that AI-EUS is non-inferior to experienced endoscopists in detecting pancreatic solid lesions [46].

### 3.2 Immune Therapeutic Response Prediction Models Based on Genetic Mutation Profiles

Recently, predicting immune treatment outcomes using tumor mutation information has become a major research direction in pancreatic cancer immunotherapy. AI networks can integrate large scale genomic data, particularly information on tumor mutation burden (TMB) and mutations in key genes, to effectively predict responses to ICIs, thereby supporting clinical decision making.

AI systems analyze genomic alteration patterns from tumor samples to detect genes linked to treatment outcomes. Studies show that changes in KMT2C and PEG3 are related to elevated tumor mutation levels and increased immune activity in pancreatic adenocarcinoma. Patients with these alterations often have an unfavorable prognosis. However, they may respond better to immune based therapies. This indicates that such genetic changes have value for predicting immune treatment response [47]. PiRNAs, as small non-coding RNAs of 18–35 nucleotides, are aberrantly expressed and highly stable in the blood of cancer patients, making them promising novel biomarkers for diagnosis and prognosis [48]. Single cell analysis and large scale transcriptome data have been used to group this disease into distinct molecular categories. These groups differ clearly in mutation patterns and in the expression levels of immune checkpoint genes. AI models can leverage these features to predict the risk of immune evasion and therapeutic responsiveness [49]. These results show that interactions between genetic alterations and local immune conditions are essential for developing accurate models to estimate immunotherapy response.

Comparisons of AI model performance between pancreatic cancer and other gastrointestinal tumors reveal that the high aggressiveness, complex TME, and low immune cell infiltration in pancreatic cancer pose significant challenges for evaluating responses to ICIs. Compared with colorectal and gastric cancers, pancreatic tumors have a lower overall tumor mutational burden. At the same time, key driver genes such as KRAS and TP53 show higher mutation frequencies. These characteristics reduce the accuracy of predictive models that rely solely on mutation data [50,51]. Moreover, the immune evasion mechanisms in pancreatic cancer are highly complex; for example, overexpression of TMEM92 has been associated with an immune tolerant phenotype, further complicating model construction [50]. 

Notably, current AI based predictive approaches remain largely in the preclinical validation stage. Most models are trained on retrospective datasets with limited sample sizes and lack prospective multicenter clinical trials to confirm applicability. Additionally, the translation of these models into clinical practice is hindered by issues such as standardized data collection of multi-omics and TME features, and the need for integration with routine clinical detection workflows. Therefore, predictive AI models for this disease should combine multiple types of information, including gene mutations, tumor immune cell patterns, checkpoint molecule levels, and local microenvironment features. Integrating these data, coupled with large scale prospective validation and standardization of detection protocols, can enhance prediction accuracy and bridge the gap between preclinical research and clinical application.

### 3.3 Multi-Omics and Machine Learning

In pancreatic cancer immunotherapy research, combining multi-omics information with machine learning has become a key approach for precision treatment. This strategy has advanced gene signature based and AI guided prognostic models. Researchers use data on gene expression (for example, the A2ML1 gene acts as a key regulatory factor, and its elevated expression is closely associated with epithelial-mesenchymal transition and tumor progression, demonstrating potential as a molecular target), DNA methylation, and mutation patterns, together with clustering and machine learning methods, to define molecular subtypes and build consensus prognostic models such as Constrained Multivariate Least Squares (CMLS) and Artificial intelligence-derived prognostic signature (AIDPS). These models allow accurate stratification of patients [52,53]. Patients with low CMLS or low AIDPS scores generally exhibit favorable prognosis, a hot tumor phenotype, abundant immune cell infiltration, and higher expression of immune checkpoint molecules, suggesting greater sensitivity to immunotherapy. In contrast, patients in the high score group have poorer prognosis, with generally higher TMB and tumor neoantigen burden (TNB), theoretically favoring immune response but also indicating more complex immune evasion mechanisms [52,53]. The AI assisted consensus prognostic model integrates multidimensional data and employs a combination of 101 machine learning algorithms to optimize predictive accuracy. It performs better than traditional single-model approaches and effectively links clinical characteristics with drug response. This provides a strong basis for designing individualized therapies for patients with pancreatic cancer [52].

In addition, spatial multi-omics approaches combined with machine learning are used to study the local immune environment in this disease. Mature tertiary lymphoid structures (TLS) are closely associated with B cell activation, immunoglobulin distribution, and extracellular matrix remodeling, serving as markers of pathological responders to immunotherapy. These studies reveal the development of tertiary lymphoid structures within tumors and show how their location relates to antitumor immune activity, which can be used to predict the efficacy of tumor immunotherapy [52]. Other researchers have also summarized the research progress and practical performance of AI in assessing Human Epidermal Growth Factor Receptor 2 (HER2)-positive pancreatic cancer status, based on pathological slides and radiomic features, as well as in supporting anti-HER2 therapies, including the prediction of treatment efficacy, recurrence, and brain metastasis risk, and facilitating the development of novel therapeutic agents [54]. AI technologies can also integrate multidimensional data to analyze complex datasets, including genomic and transcriptomic profiles, thereby optimizing the detection of biomarkers such as circulating tumor DNA (ctDNA) and microRNAs (miRNAs), and enabling early diagnosis, prognostic prediction, and treatment response monitoring in pancreatic cancer [4].

**Figure 2:**
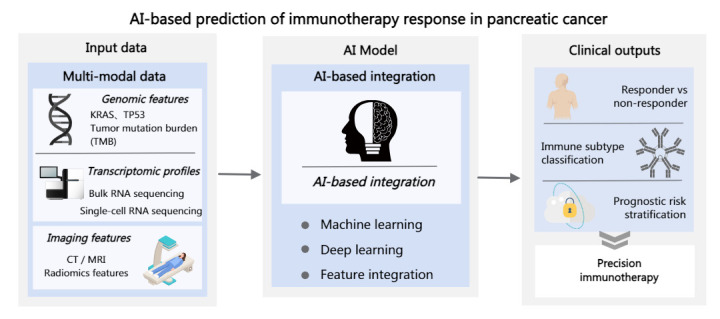
**AI based prediction of immunotherapy response in pancreatic cancer.** AI integrates multi-modal data (genomic alterations, transcriptomic profiles, and imaging features) via machine learning and deep learning; its models further classify patients into responders/non-responders, identify immune subtypes, and conduct prognostic risk stratification, thus enabling precision immunotherapy (The figures were created using MedPeer).

These findings suggest that AI based spatial transcriptomic analysis can accurately identify patients who respond to immunotherapy, thereby optimizing clinical decision making [52]. High throughput metabolite quantification combined with unsupervised learning allows for precise stratification of pancreatic cancer subtypes, prediction of treatment response and survival, and facilitates the development of metabolism targeted therapies [53] (Fig. 2).

## 4 Application of Artificial Intelligence in Clinical Decision Making for Pancreatic Cancer Immunotherapy

### 4.1 AI Assisted Dynamic Monitoring of Treatment Response

In the immunotherapy regimen for pancreatic cancer, dynamic monitoring of treatment response is crucial for evaluating efficacy during therapy and making timely adjustments to treatment strategies. 

First, real time assessment of therapeutic efficacy based on multi-modal data is central to AI assisted dynamic monitoring. Traditional single modality approaches struggle to fully capture the dynamic changes of pancreatic tumors. AI techniques, through deep learning and machine learning models, can analyze data from circulating tumor DNA, protein biomarkers, and imaging examinations such as CT, MRI, and endoscopic ultrasound in real time, enabling dynamic monitoring of tumor burden and immune activity. For instance, combining information from biopsy guided by endoscopic ultrasound with imaging data allows AI to increase early detection accuracy. It also helps assess how tumors respond to immunotherapy [55]. In addition, AI models that integrate genetic mutation data with measures of immune cell infiltration can estimate how well patients respond to immune checkpoint therapy. They also help identify those individuals who are most likely to benefit from treatment [26].

**Figure 3:**
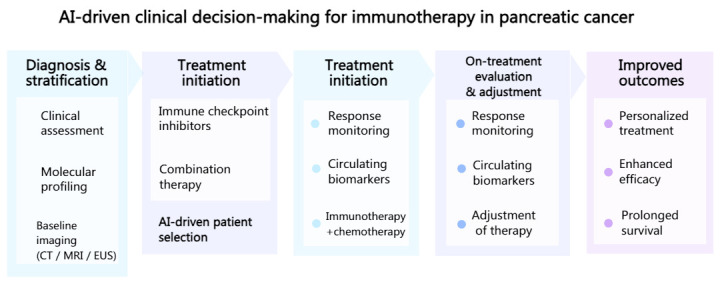
**AI assisted clinical decision making for immunotherapy in pancreatic cancer.** AI supports clinical decision making throughout the immunotherapy process in pancreatic cancer. Starting from diagnosis and patient stratification based on clinical, molecular, and imaging data, AI assists in treatment selection and initiation. During therapy, continuous monitoring of treatment response and circulating biomarkers enables dynamic adjustment of therapeutic strategies. This AI assisted approach facilitates personalized treatment, improved therapeutic efficacy, and prolonged patient survival (The figures were created using MedPeer).

Second, the integrated analysis of biomarkers and imaging data is key to achieving precise dynamic monitoring. AI uses multi-level data fusion techniques to combine spatial single cell sequencing data of tumor tissue with radiomic features, revealing tumor heterogeneity and dynamic changes in immune cell distribution. This approach helps identify novel predictive biomarkers and guides adjustments to personalized treatment strategies [27]. In addition, AI models can dynamically integrate patient clinical data, treatment regimens, and efficacy indicators to build individualized frameworks for predicting treatment response. This not only improves the accuracy of efficacy assessment but also facilitates personalized management and dynamic adjustment of immunotherapy [25] (Fig. 3). In the future, as data collection methods and AI techniques continue to improve, combining multiple types of data is likely to become standard for continuous monitoring of immunotherapy in this disease. This approach will support more accurate individualized treatment and help enhance both patient survival and wellbeing.

### 4.2 AI Assisted Clinical Stratification and Precision Therapy

The application of AI in clinical classification and individualized therapy for pancreatic cancer focuses on integrating patient characteristics, genomic information, and immune cell patterns to achieve precise risk assessment. This approach enables the optimization of immunotherapy, reduces ineffective treatments, and improves both patient survival and wellbeing. Due to the high heterogeneity of pancreatic tumors and their complex microenvironment, conventional treatment and risk stratification methods have notable limitations. By employing deep learning and multi-modal data integration, AI provides a promising strategy for more accurate patient grouping and personalized treatment planning. 

AI technology can integrate patients’ clinical information, imaging data, and genomic features to construct multidimensional risk assessment models. For instance, studies using the tumor–stroma ratio have found a strong link between TSR and patient prognosis in this disease. Individuals with a low TSR tend to have better outcomes and may respond more effectively to chemotherapy combining gemcitabine with albumin bound paclitaxel. By applying AI assisted deep learning models to automatically analyze pathological slides and imaging data, TSR can be accurately evaluated, enabling patient stratification and the development of precise treatment plans [56]. In addition, AI assisted quantification of tumor budding, through digital image processing and automated recognition, has been shown to correlate significantly with pancreatic cancer aggressiveness and patient survival, further supporting the value of AI in patient risk stratification [57].

In terms of genomic and immune features, AI combined with radiomics and liquid biopsy technologies enables noninvasive early diagnosis and prediction of genetic mutations such as KRAS and TP53 in pancreatic cancer patients, enhancing individualized clinical management. Studies indicate that radiomics models combined with liquid biopsy information can accurately detect KRAS mutations in this disease, achieving an area under the curve of 0.97. These models provide strong evidence for predicting patient response to immunotherapy [58]. Meanwhile, AI assisted multi-cancer predictive models that integrate electronic health records improve the specificity of pancreatic cancer risk prediction, reduce misdiagnosis caused by confounding factors, and thereby optimize patient screening and treatment decision making [59].

For precise immunotherapy in this disease, AI helps determine which patients may benefit from immune checkpoint inhibitors. It also allows continuous monitoring of treatment effects by analyzing features of the immune environment within tumors. This information can guide adjustments to therapy regimens. For example, AI based radiomics and multi omics data integration can reveal immune cell infiltration patterns and immune escape mechanisms within tumors, providing precise biomarkers and risk stratification strategies for individualized immunotherapy [25]. In addition, AI has demonstrated significant value in body composition analysis during the clinical treatment of pancreatic cancer patients. By automatically assessing sarcopenia from CT images, AI models achieve high accuracy in muscle and adipose tissue segmentation, offering quantitative evidence for prognosis evaluation and nutritional intervention during treatment [60].

### 4.3 AI Assisted Neoadjuvant Therapy and Conversion Surgery

Neoadjuvant therapy (NAT) is an important component of pancreatic cancer treatment. By applying systemic therapy before surgery, NAT reduces tumor burden, such as downstaging TNM classification and grade and decreasing tumor volume, thereby improving resectability and enhancing patient survival outcomes. AI in this area is mainly used to evaluate treatment effectiveness, support surgical planning, and predict patient risk. These capabilities help implement tailored therapy strategies for individual patients. 

AI has demonstrated significant potential in predicting responses to neoadjuvant immunotherapy and assessing surgical feasibility. Assessing the remaining tumor tissue after preoperative therapy in this disease remains difficult using conventional pathological methods. In contrast, deep learning algorithms applied to digital processing and automated segmentation of histopathological images from resected specimens can achieve high accuracy in classifying tumor tissue, normal ducts, and residual epithelium. Notably, the F1 score for tumor tissue classification reached 0.86, with an overall multi class average score of 0.82, demonstrating the feasibility and accuracy of AI based approaches for objective assessment of neoadjuvant treatment efficacy [61,62]. Moreover, convolutional neural network models based on preoperative CT images can predict pathological responses to neoadjuvant therapy. When combined with changes in the serum biomarker CA19-9, these models achieved high predictive accuracy with an AUC of 0.785, outperforming predictions based on imaging or biomarkers alone [63].

Imaging plays a central role in AI assisted surgical decision making, risk assessment, and treatment response prediction. Imaging methods such as CT and MRI are commonly used to evaluate and stage pancreatic ductal adenocarcinoma. However, small metastases and tissue changes after preoperative therapy are difficult to assess accurately. AI assisted diagnostic tools can enhance early tumor detection and improve evaluation of blood vessel invasion and lymph node spread. This supports better selection of patients for surgery [64,65]. Particularly in conversion surgery, AI integrated with biomarkers and multi modal imaging helps evaluate tumor resectability and recurrence risk, enabling individualized preoperative risk stratification and outcome prediction. This multidisciplinary approach, supported by AI assisted precise data analysis, improves the success rate of conversion surgery and enhances postoperative survival quality. In addition, digital pathology combined with spatial point analysis using AI reveals how preoperative therapy affects the immune environment within tumors. Notably, it shows a closer spatial relationship between CD8 positive T cells and cancer cells, which is linked to longer survival after surgery. These findings suggest that AI not only facilitates treatment response assessment but also contributes to prognosis prediction and monitoring of immunotherapy efficacy [66].

Currently, technologies such as AI assisted imaging diagnosis, dynamic efficacy monitoring, and AI aided clinical stratification have gradually become applicable in clinical practice. In contrast, applications including AI assisted multi-omics response prediction, spatial analysis of the immune microenvironment, and dynamic treatment optimization decision making still require more future research and trials to verify their effectiveness and safety.

## 5 Current Challenges and Future Directions

### 5.1 Data Quality and Integration

A major challenge for using AI in pancreatic cancer immunotherapy is the heterogeneity and lack of standardization in training datasets. Addressing this issue is essential for reliable model development. Due to substantial variations in clinical presentation, TME, and genetic mutation profiles among pancreatic cancer patients, training datasets are often highly heterogeneous, which compromises the generalizability and robustness of AI models and limits their practical clinical implementation. To enhance model robustness, rigorous data standardization is required, including harmonization of data acquisition protocols, imaging annotation standards, and preprocessing methods for genomic data [67]. For instance, a review and meta analysis of patients with gastrointestinal tumors receiving immunotherapy found that AI models using genetic mutation data accurately predicted treatment responses. However, the AUC for pancreatic cancer was markedly lower than that for gastric cancer, with AUC values of 0.52 and 0.87, respectively. This discrepancy highlights the significant impact of data heterogeneity and insufficient standardization on model performance [26]. Thus, developing consistent standards for data collection and processing is essential to improve how accurately AI can predict responses in pancreatic cancer immunotherapy.

In addition, the integration of large scale, multi-center data is also crucial for promoting the clinical translation of AI models. Data from a single center are often limited in sample size and may exhibit regional and population biases, making them insufficient for training complex models. By integrating clinical and genomic data from multiple centers, researchers can not only expand the sample size and enhance the representativeness and stability of the models, but also encompass a more diverse range of patient characteristics, thereby improving model generalizability. For example, using expression data from ten multi-center cohorts comprising a total of 1280 patients, researchers successfully identified 32 prognosis related genes and constructed an AI derived pancreatic cancer prognostic signature (AIDPS) through machine learning. This model performed excellently in three independent validation cohorts totaling 290 patients, demonstrating strong predictive power and clinical utility [68]. Furthermore, multi-center data sharing facilitates model validation across different clinical settings, helping to identify potential biases and limitations, and promoting continuous model optimization and refinement.

### 5.2 Clinical Translation of AI Models

The clinical translation of AI models from research to practical application still faces multiple challenges. First, there is a difficult to reconcile conflict between model interpretability and clinical acceptability. The trust of clinicians and patients in AI models largely depends on their transparency and explainability. Many current deep learning models achieve high accuracy but operate as “black boxes”. Clinicians often cannot see how decisions are made, which limits their use in clinical practice. For example, in pancreatic cancer imaging diagnosis and immunotherapy response prediction, although models can efficiently analyze complex imaging or genomic data, the lack of clear biological explanations restricts their clinical implementation [69].

Second, regulatory, ethical, and patient privacy issues pose unavoidable challenges for the clinical application of AI. Currently, the regulatory framework for AI based medical devices is not fully mature, especially in the emerging field of tumor immunotherapy, with a lack of specific standards and guidelines, making it difficult for AI products to obtain regulatory approval [70]. From an ethical viewpoint, heavy use of patient data in AI training raises concerns about privacy and security. Sensitive patient details, such as genomic profiles, imaging files, and electronic health records, must be carefully protected during collection, storage, and use to avoid breaches [71]. In addition, AI algorithms may exhibit data bias and fairness issues; if the training data lack diversity, the model may produce flawed or biased diagnostic and treatment recommendations for certain patient groups [72].

### 5.3 AI Assisted Immunotherapy

Due to its pathological characteristics, including low TMB, a highly immunosuppressive TME, and a dense stromal barrier, pancreatic cancer poses significant challenges for AI based prediction of immunotherapy efficacy. Existing AI models that rely solely on genomic mutation data show limited predictive accuracy. Therefore, a comprehensive elucidation of immune evasion mechanisms in pancreatic cancer, together with deep integration of multi-omics data, is urgently needed to enhance the multimodal data fusion capability of current models, thereby improving immunotherapy response prediction and enabling more precise treatment strategies. With the continuous advancement of immunotherapy research in pancreatic cancer, gene editing technologies and novel cell therapies have become key focuses of innovation. AI, as a powerful tool for data analysis and pattern recognition, is increasingly applied to assist in the design and optimization of these new strategies. Gene editing technologies, particularly the CRISPR/Cas9 system, are gaining prominence in pancreatic cancer immunotherapy. AI algorithms can optimize the selection of gene editing targets, predict editing efficiency and off-target risks, thereby enhancing the safety and efficacy of gene therapy. Using AI to analyze single cell sequencing data from both tumor and immune cells allows detailed characterization of tumor heterogeneity. This information can guide precise gene editing to improve antigen presentation or reduce immunosuppression. The combination of gene editing and AI holds promise for developing personalized immunotherapy approaches to overcome immune tolerance in pancreatic cancer [73]. In the field of novel cell therapies, such as chimeric antigen receptor T cell (CAR-T) therapy, tumor infiltrating lymphocyte (TIL) therapy, and genetically modified natural killer (NK) cell therapy, AI plays a pivotal role in cell design, target selection, and efficacy prediction. By applying machine learning models to patient genomic, proteomic, and immunophenotypic data, AI can identify potential targets and predict responses to cell therapy as well as possible immune related adverse effects, guiding individualized treatment adjustments. Additionally, AI assists in optimizing cell preparation processes and quality control, improving the safety and consistency of cell based therapies [74].

Pancreatic cancer is a highly aggressive tumor in the clinical setting, and its immunotherapy faces numerous challenges, including a complex TME and immune tolerance, which greatly limit improvements in patient prognosis and quality of life. In recent years, rapid advances in AI have created new opportunities for progress in this field. By integrating multi-modal data, AI can deeply dissect the heterogeneity of the pancreatic cancer immune microenvironment, providing a solid theoretical and technological foundation for the development of more precise, effective, and personalized immunotherapy strategies.

In current research on pancreatic cancer immunotherapy, the unstable immune tolerance caused by the complex and heterogeneous TME is a key barrier limiting clinical efficacy. AI assisted technologies can optimize the selection of immune related biomarkers and enable early diagnosis, thereby facilitating accurate prediction of immunotherapy responses. With the introduction of AI, particularly the development of clinical decision support systems, clinicians are now equipped with tools for immunotherapy monitoring and patient stratification, further advancing clinical translation and precision medicine.

However, the application of AI in pancreatic cancer immunotherapy still faces numerous challenges. First, issues related to data standardization, high quality data collection, and data integration limit the generalizability and clinical applicability of AI models. Second, the black box nature of AI models affects their interpretability and trustworthiness in clinical settings, restricting acceptance by clinicians and patients. At the same time, the role of AI as a tool for data analysis and pattern recognition in the design and optimization of immunotherapy strategies remains to be further explored.

Looking ahead, advancing pancreatic cancer immunotherapy toward precision and intelligence requires emphasis on large scale, multi center clinical validation to ensure the safety and effectiveness of AI assisted systems. At the same time, with the continuous improvement of relevant regulations, data privacy protection and ethical oversight will provide a solid foundation for the clinical application of AI technologies. In addition, accurately addressing patients’ individual needs will be a key focus of future research. Only by fully considering patients’ genetic background, immune status, and lifestyle can truly personalized treatment plans be developed and optimized.

## 6 Conclusion

In summary, the field of pancreatic cancer immunotherapy is at a critical juncture, transitioning from traditional experience based approaches toward intelligent and precision strategies. Artificial intelligence, as a core driving force, is leveraging its powerful data processing and pattern recognition capabilities to advance in-depth understanding of the TME and foster innovation in immunotherapy strategies. By balancing diverse research perspectives and integrating interdisciplinary technologies, AI is poised to become an indispensable support in pancreatic cancer immunotherapy, significantly improving clinical outcomes and patient survival quality.

## Data Availability

The data supporting the findings of this study are available from the corresponding author upon reasonable request.
